# IGF-1 Increases Collagen Deposition by Dermal Fibroblasts: Applications for Tissue Engineering

**DOI:** 10.3390/cells15111023

**Published:** 2026-06-02

**Authors:** David Brownell, Alexane Thibodeau, Guillaume Locatelli, Aiden Smith, Megan Richer, Stéphane Chabaud, Stéphane Bolduc

**Affiliations:** 1 Centre de Recherche en Organogénèse Expérimentale/LOEX, CHU de Québec-Université Laval Research Center (Regenerative Medicine Division), Université Laval, Quebec City, QC G1J 4A4, Canada; david.brownell@crchudequebec.ulaval.ca (D.B.); alexane.thibodeau.1@ulaval.ca (A.T.); aiden.smith.1@ulaval.ca (A.S.); megan.richer@crchudequebec.ulaval.ca (M.R.); stephane.chabaud@crchudequebec.ulaval.ca (S.C.); 2 École Supérieure de Physique et de Chimie Industrielles de la Ville de Paris (ESPCI Paris), Université Paris Sciences et Lettres (PSL), 75005 Paris, France; 3 Department of Surgery, Université Laval, Quebec City, QC G1V 0A6, Canada

**Keywords:** insulin growth factor, tissue engineering, extracellular matrix, dermis

## Abstract

**Highlights:**

**What are the main findings?**

- IGF-1 increased collagen deposition and improved mechanical properties of dermis reconstructed using the self-assembly approach.
- Decrease in collagen synthesis due to aging can be reversed in vitro by the use of IGF-1.

**What are the implications of the main findings?**

- ECM produced by fibroblasts can be increased by the use of IGF-1 to reach mechanical properties adequate for surgical manipulation.
- Use of IGF-1 during reconstruction of dermis from patients 50 years and older paves the way to produce completely biological midurethral slings.

**Abstract:**

Tissue engineering using the self-assembly approach represents a promising technology. However, age-related reductions in extracellular matrix deposition by stromal cells limit the mechanical robustness of reconstructed tissues what can be critical for midurethral sling reconstruction. Indeed, stress urinary incontinence predominantly affects women over 50 years of age and is commonly treated by implantation of midurethral slings, whose synthetic versions have raised concerns regarding safety and long-term tolerance. In this study, we investigated whether biochemical modulation could enhance collagen deposition and mechanical properties of self-assembled dermal tissues reconstructed from female donors of different ages. Dermal fibroblasts were cultured in the presence of ascorbic acid, and the effects of hormonal supplementation, metabolic and hypoxia-related stimuli, and insulin signaling activation were evaluated using collagen quantification, histological analyses, and mechanical testing. Fibroblasts derived from younger donors deposited significantly more collagen than those from older female donors. Among all tested conditions, insulin like growth factor 1 (IGF 1) markedly increased collagen deposition in a dose-dependent manner, including in fibroblasts from women over 50 years of age, whereas β-estradiol and progesterone had no significant effect on collagen content. Although β-estradiol slightly increased tissue thickness, only IGF-1 supplementation resulted in substantial improvements in perforation strength, stiffness, displacement at break, and toughness. These results demonstrate that IGF-1 is a potent enhancer of extracellular matrix production and mechanical performance in dermal tissues reconstructed by the self-assembly approach, and represents a promising strategy to improve the development of biological midurethral slings.

## 1. Introduction

Tissue engineering offers a promising approach to in vitro tissue development [1,2,3]. While most strategies rely on biomaterial scaffolds, these constructs often fail to recapitulate the structural and functional complexity of native tissues and may induce inflammatory or inappropriate cellular responses. To overcome these limitations, tissue engineering approaches based on self-assembly enable stromal cells to produce and organize their own extracellular matrix (ECM), resulting in living tissues that closely resemble native human counterparts [4]. This approach has been successfully applied to the reconstruction of several tissues, including skin, blood vessels, and genitourinary tissues, and has supported both translational and fundamental studies [4].

The mechanical strength of the reconstructed tissues emerged as a critical limitation, particularly when dermal fibroblasts derived from women over 50 years of age were used. These fibroblasts produce lower amounts of collagen, a phenomenon likely related not only to cellular aging but also to the complex hormonal changes associated with menopause [5,6].

As an example of a condition affecting mainly postmenopausal women, stress urinary incontinence (SUI) is a major urological condition affecting up to one woman out of three over the course of their lifetime [7,8]. Its main risk factors include multiparity, vaginal trauma, and obesity, all of which contribute to pelvic floor weakening [9].

When conservative management fails, surgical intervention is required to restore urethral support, most commonly through the implantation of a mid-urethral sling (MUS) [10,11]. Although synthetic MUS have been widely used, they have been associated with significant complications in a subset of patients, including chronic pain and vaginal or urethral erosion, sometimes necessitating device removal [12,13]. These concerns ultimately led to the withdrawal of several synthetic slings from the market and their ban by the U.S. Food and Drug Administration (FDA) [14], highlighting the urgent need for safer and more biologically relevant alternatives [15]. Biological MUS reconstructed using the self-assembly technique have therefore been explored as an alternative to synthetic devices. While initial results were encouraging, the age- and hormone-dependent decline in ECM deposition represents a major obstacle for the development of mechanically robust tissue-engineered MUS intended for the population most affected by SUI.

Beyond classical biochemical supplements [16,17] and mechanical stimulation [18], increasing interest has focused on key regulators of stromal metabolism and ECM synthesis. Estrogen supplementation has been reported to stimulate collagen production and matrix deposition in fibroblasts, supporting its potential relevance in postmenopausal tissue engineering strategies [19]. In parallel, metabolic modulators such as insulin [20], sometimes associated with hypoxia, and adenosine [21,22] have been shown to enhance ECM synthesis through anabolic and stress-responsive signaling pathways, although their impact on the mechanical properties of tissues reconstructed using the self-assembly approach has yet to be characterized and variable across donors.

Insulin-like growth factor-1 (IGF-1), a major downstream mediator of insulin signaling, is a potent anabolic regulator of connective tissues. IGF-1 promotes fibroblast proliferation, collagen synthesis, and ECM organization, and plays a key role in tissue growth and repair [23,24]. In contrast to insulin, IGF-1 primarily targets stromal cells through IGF-1R signaling and ECM-associated mechanisms, making it particularly attractive for strategies aiming to enhance endogenous matrix production [25,26,27]. Among its variants, LONG^®^ R^3^ IGF-I (IGF-1 LR), a long-acting analog with enhanced stability and bioavailability [28,29,30], may allow prolonged activation of IGF-1 signaling during tissue reconstruction, and thereby, improve collagen deposition and structural integrity.

Based on these observations, we hypothesized that supplementation of self-assembly culture conditions with IGF-1 LR, alone or in combination with estrogen, would enhance extracellular matrix deposition, particularly collagen synthesis, and improve the mechanical properties of engineered tissues. The objectives of this study were threefold: (i) to characterize inter-donor variability in collagen deposition among dermal fibroblasts derived from multiple female donors under standard conditions; (ii) to evaluate the effects of IGF-1 LR and estrogen, alone or in combination, on ECM deposition; and (iii) to determine whether these biochemical modulations translate into improved mechanical properties of tissues reconstructed using the self-assembly approach.

## 2. Materials and Methods

### 2.1. Ethics

This study was conducted in accordance with the Declaration of Helsinki and was approved by the institutional committee for the protection of human participants (Comité d’éthique de la recherche du CHU de Québec–Université Laval, protocol number DR-002-1190). All donors provided written informed consent prior to biopsy collection.

### 2.2. Cell Culture

#### 2.2.1. Dermal Fibroblast Extraction from Skin Biopsies 

Dermal biopsies were obtained from eight healthy female donors undergoing plastic surgery. Protocol has been previously described [31]. Briefly, specimens were rinsed five times in phosphate-buffered saline (PBS) containing 100 U/mL penicillin (Sigma-Aldrich, St. Louis, MO, USA), 25 µg/mL gentamicin (Schering, Pointe-Claire, QC, Canada), and 0.5 µg/mL amphotericin B (Fisher Scientific, Ottawa, ON, Canada). Biopsies were cut into small strips and incubated overnight at 4 °C in a thermolysin solution (500 µg/mL; Sigma-Aldrich, St. Louis, MO, USA) diluted in 25 mM HEPES buffer (MP Biomedicals, Montreal, QC, Canada) containing 1 mM CaCl_2_ (Sigma-Aldrich, St. Louis, MO, USA) (pH 7.4) to digest the basal lamina. The epithelium was manually separated from the stroma using tweezers. Stromal tissues were then incubated for 3 h in a collagenase H solution (125 U/mL; Boehringer Mannheim, Laval, QC, Canada) diluted in Dulbecco’s Modified Eagle’s Medium (DMEM, Corning, Corning, NY, USA) supplemented with 10% fetal bovine serum (FBS); Avantor Seradigm FB Essence, Radnor, PA, USA), 100 U/mL penicillin, and 25 µg/mL gentamicin) at 37 °C in a humidified incubator with 8% CO_2_. Fibroblasts were collected by centrifugation and cryopreserved in FBS containing 10% dimethyl sulfoxide (DMSO; Sigma-Aldrich, St. Louis, MO, USA) until further use.

#### 2.2.2. Dermal Fibroblast Expansion 

Dermal fibroblasts were cultured in DMEM/10%FBS + antibiotics until reaching confluence, then either passaged or used for tissue reconstruction.

### 2.3. Dermis Reconstruction Using the Self-Assembly Technique 

Dermis were produced as previously described [17]. Briefly, dermal fibroblasts at passage 3 were seeded at a density of 5 × 10^4^ cells/cm^2^ in six-well plates (Falcon, Corning, NY, USA). A ring (outer diameter 33 mm, inner diameter 25 mm) made of Whatman qualitative filter paper (Grade 4, GE Healthcare, Little Chalfont, UK) was placed at the bottom of each well to facilitate sheet handling and to limit tissue contraction. Filter paper rings were secured using custom-made stainless-steel air–liquid support rings (outer diameter 31.5 mm, inner diameter 29 mm, height 5 mm with six equidistant 2.5 mm-height gates, alternating from top to bottom positions, allowing culture media circulation). Cells were cultured in DMEM/10%FBS + antibiotics supplemented with 50 µg/mL 2-phospho-L-ascorbic acid trisodium salt (2PAA; Sigma-Aldrich, St. Louis, MO, USA) [32]. Culture medium was changed three times per week. Cultures were maintained for 10 days for collagen deposition quantification or for 28 days for stroma reconstruction. Beta-estradiol (Sigma-Aldrich, St. Louis, MO, USA), progesterone (Sigma-Aldrich, St. Louis, MO, USA), cobalt chloride (Sigma-Aldrich, St. Louis, MO, USA) [33], adenosine (Sigma-Aldrich, St. Louis, MO, USA), insulin (Sigma-Aldrich, St. Louis, MO, USA), IGF-1 LR3 (Gibco, Thermo Fisher Scientific, Waltham, MA, USA) were added at the indicated concentrations when specified. Based on preliminary experiments and to ensure robust responses in long-term cultures, 1 µg/mL was used in selected experiments despite dose–response results indicating efficacy at 0.5 µg/mL.

### 2.4. Collagen Deposition Quantification 

Total collagen and non-collagenous protein contents were quantified using a Sirius Red/Fast Green collagen staining kit (Chondrex, Woodinville, WA, USA) according to the manufacturer’s protocol. Absorbance was measured using a SpectraMax Plus spectrophotometer (Molecular Devices, San Jose, CA, USA) at 540 and 605 nm.

### 2.5. Total DNA Quantification 

Tissue samples were homogenized in 200 µL PBS containing 1.6 U/mL proteinase K (Bio Basic, Markham, ON, Canada) and incubated overnight at 56 °C after adjustment of the volume to 210 µL with 10 mM Tris-HCl (Sigma-Aldrich, St. Louis, MO, USA) (pH 7.5) and 10 mM EDTA (Bio Basic, Markham, ON, Canada). Proteinase K was then inactivated at 75 °C for 20 min. A 63 µL aliquot of the digest was incubated with 3 µL of RNase solution (20 mg/mL; Invitrogen, Waltham, WA, USA) for 2 h at 37 °C. Samples were diluted with 54 µL of 10 mM Tris-HCl (pH 7.5), 1 mM EDTA. DNA content was quantified using the Quant-iT PicoGreen dsDNA Assay Kit (Invitrogen, Eugen, OR, USA) according to the manufacturer’s instructions. Measurements were performed in technical triplicates using independent tissue samples.

### 2.6. Thickness Measurement 

The thickness of stromal constructs was measured using a non-contact laser thickness gauge (Keyence, Osaka, Japan). Measurements were performed on hydrated tissues to preserve their native structure. Each construct was positioned flat on the measurement platform, and thickness was determined at two locations across the surface of each stroma. The mean value for each sample was calculated and used for subsequent analyses.

### 2.7. Mechanical Testing 

For each condition, four reconstructed single-layer tissues (33 mm diameter; generated in 6-well plates) were tested. Puncture testing was performed using an ElectroPuls E1000 mechanical testing system (Instron, Norwood, MA, USA) equipped with dedicated puncture fixtures and a 3 mm diameter spherical probe (similar to a pinhead) attached to the moving crosshead. Force acquisition was achieved using a ±10 N load cell with a sensitivity of ±2.5 mN.

Samples were carefully positioned flat over a metallic holder containing a central circular aperture (3 mm diameter; Biomomentum Inc., Laval, QC, Canada). The probe was aligned with the center of the construct and displaced vertically at a constant rate of 1.2 mm·min^−1^ until perforation.

Throughout the assay, force and displacement were continuously recorded to generate force–displacement and strain curves for each sample. Results are presented as mean ± standard deviation, and graphs were produced using GraphPad Prism v.9.2 (San Diego, CA, USA).

Maximum strength (mN) was defined as the highest force recorded prior to abrupt tissue failure. Stiffness (mN·mm^−1^) was calculated from the steepest slope of the force–displacement curve. Toughness (mJ) corresponded to the area under the curve from the start of the test up to failure, reflecting the total energy required to induce rupture or puncture. Displacement (mm) was defined as the displacement from the beginning of the test to failure.

### 2.8. Histological Staining 

Reconstructed dermal tissues were fixed in 3.7% formaldehyde, embedded in paraffin, sectioned (6 µm), and stained with Masson’s trichrome. The thickness of the stroma was assessed using a Zeiss Axio Imager M2 microscope equipped with an AxioCam HR Rev3 camera (Carl Zeiss, Oberkochen, Germany). Images were processed with the AxioVision 40 V4.8.2.0 software (Carl Zeiss, Oberkochen, Germany), and scale bars were added with ImageJ 1.53e software (NIH, Bethesda, MD, USA).

### 2.9. Statistical Analysis 

Statistical analyses were performed using GraphPad Prism software version 9.2 (San Diego, CA, USA). Data are presented as mean ± standard deviation. One-way analysis of variance (ANOVA) followed by appropriate post hoc tests (see figure legends) was used to compare experimental groups. A *p*-value < 0.05 was considered statistically significant.

## 3. Results

### 3.1. Age of Donors Influenced the Amount of Deposited Collagen in Dermis Reconstructed by the Self-Assembly Method

**Figure 1:**
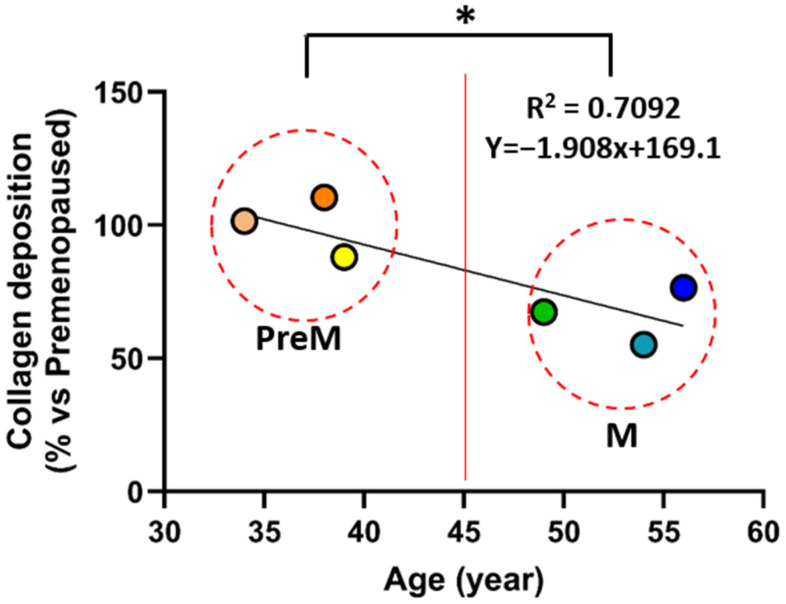
**Inter-donor variability in collagen deposition by dermal fibroblasts as a function of donor age.** Collagen deposition by dermal fibroblasts isolated from female donors of different ages and cultured in the presence of ascorbic acid. Total collagen content was quantified after 10 days of culture using Sirius Red/Fast Green staining. Donors were grouped as premenopaused (PreM) donor-derived fibroblasts fibroblasts (≤45 years: 34, 38 and 39 years old) and Postmenopausal (M) donor-derived fibroblasts (>45 years: 49, 54 and 56 years old). A significant decrease in collagen deposition was observed in fibroblasts derived from Postmenopausal donors compared to premenopaused donors. Each point represents an individual donor. Data are presented as mean (N = 3 donors per group, n = 3). Statistical significance was assessed using an unpaired *t*-test. Statistical significance was indicated by an asterisk * (*p* < 0.05). Color code: F34 ● F38 ● F39 ● F49 ● F54 ● F56 ●.

Dermal fibroblasts were cultured in the presence of ascorbic acid to promote extracellular matrix deposition, and total collagen production was quantified. Fibroblasts were isolated from female donors and divided into two groups based on donor age: premenopaused donor-derived fibroblasts (<45 years; 34, 38 and 39 years old; mean age 37 ± 2.6 years) and Postmenopausal donor-derived fibroblasts (>45 years; 49, 54 and 56 years old; mean age 53 ± 3.6 years). Dermal fibroblasts from premenopaused donors deposited significantly more collagen compared to fibroblasts from Postmenopausal donors (18.67 µg/cm^2^ ± 2.52 (or 100% of PreM average) vs. 12.17 µg/cm^2^ ± 1.76 (or 65.2% of PreM average), respectively; N = 3 donors per group; *p* = 0.026). Linear regression analysis showed a significant decrease in collagen deposition with age (slope = −1.91, R^2^ = 0.71, *p* = 0.035) (Figure 1).

### 3.2. IGF-1 Increased the Amount of Deposited Collagen in Dermis Reconstructed by the Self-Assembly Method

As stress urinary incontinence mainly affects women over 50 years of age, reinforcing extracellular matrix deposition by dermal fibroblasts derived from this population is essential to improve the mechanical performance and clinical relevance of engineered tissues. Several biochemical factors were therefore screened for their ability to enhance collagen deposition.

**Figure 2:**
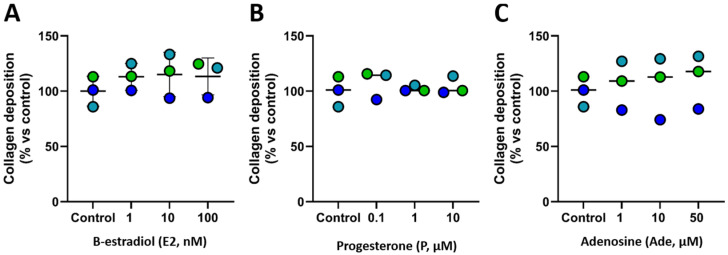
**Screening of biochemical factors influencing collagen deposition by dermal fibroblasts.** Effect of various biochemical supplements on collagen deposition by dermal fibroblasts cultured using the self-assembly approach. Fibroblasts were treated with increasing concentrations of (**A**) β-estradiol (E2) and (**B**) progesterone (P), and (**C**) adenosine (Ade). Total collagen content was quantified using Sirius Red staining and normalized to control conditions. N = 3, n = 3. Values are expressed as % of the untreated controls. Statistical analyses were performed using ANOVA one-way followed by Dunnett’s multiple comparison test. Data represent mean values from independent experiments. Non-significant differences (*p* ≥ 0.05) are not indicated. Color code: F49 ● F54 ● F56 ●.

First, β-estradiol (Figure 2A) and progesterone (Figure 2B) were added to fibroblast cultures at various concentrations (Figure 2A), but no significant increase in collagen deposition was observed. Purinergic-related pathways were then investigated by supplementing cultures with adenosine. Adenosine did not significantly affect collagen deposition (Figure 2C). Stimulation of hypoxia-related pathways were also tested by the addition of cobalt chloride (CoCl_2_) but chemical induction of hypoxia led to a marked reduction in collagen production (Figure S1).

**Figure 3:**
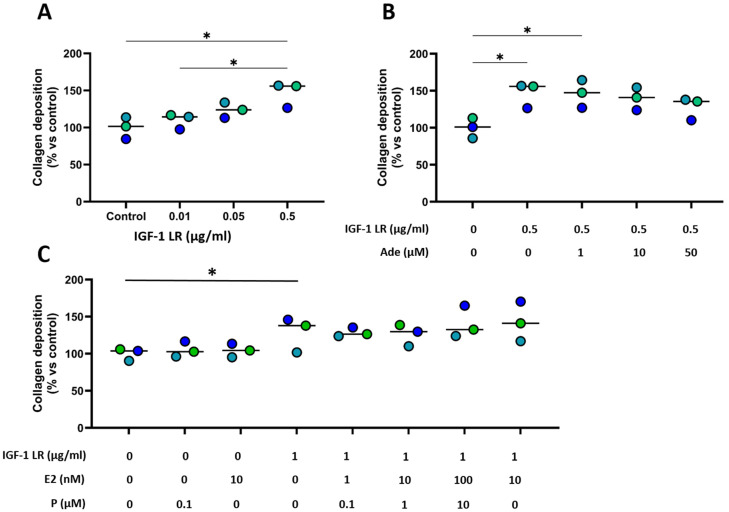
**Screening of biochemical factors influencing collagen deposition by dermal fibroblasts.** Effect of various biochemical supplements on collagen deposition by dermal fibroblasts cultured using the self-assembly approach. Fibroblasts were treated with increasing concentrations of (**A**) IGF-1 LR3 (IGF-1 LR), (**B**) combined treatments with IGF-1 LR3 (IGF-1 LR) and adenosine (Ade) (**C**) or combined treatments with IGF-1 LR3 (IGF-1 LR), and/or beta-estradiol (E2) and/or progesterone (P) were also evaluated. Total collagen content was quantified using Sirius Red staining and normalized to control conditions. No significant differences were observed between IGF-1 LR alone and combination treatments. (IGF-1 LR + adenosine, E2, or P). N = 3 (F49, F54, F56), n = 3. Values are expressed as percentage of untreated controls. Statistical analyses were performed using ANOVA one-way followed by Tukey’s multiple comparison test. Data represent mean values from independent experiments. Statistical significance was indicated by an asterisk * (*p* < 0.05). Non-significant differences (*p* ≥ 0.05) are not indicated. Color code: F49 ● F54 ● F56 ●.

Next, stimulation of the insulin signaling pathway using IGF-1 LR3 resulted in a significant, dose-dependent increase in collagen deposition (Figure 3A). Combined treatments with IGF-1 LR3 and β-estradiol, progesterone, or both hormones did not further enhance collagen deposition compared to IGF-1 LR3 alone (Figure 3B). Finally, the addition of adenosine to IGF-1 LR3 –supplemented cultures led to a progressive, but not statistically significant, decrease in collagen deposition with increasing adenosine concentrations (Figure 3C).

### 3.3. IGF-1 Increased the Amount of Collagen but Also the Thickness of Reconstructed Dermis Regardless the Donors’ Age

**Figure 4:**
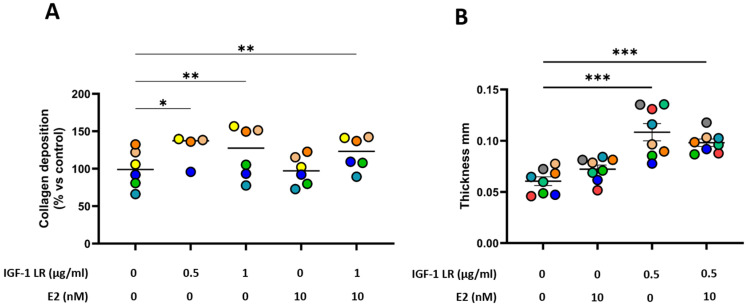
**IGF-1 LR and β-estradiol increase collagen deposition and dermal thickness.** Effects of IGF-1 LR3 (IGF-1 LR), alone or in combination with β-estradiol (E2), on extracellular matrix production in reconstructed dermal tissues. No significant differences were observed between IGF-1 LR alone and combination treatments. (**A**) Total collagen content quantified after 28 days of tissue reconstruction using Sirius Red/Fast Green staining. (**B**) Dermal thickness measured on histological sections. Each colored symbol represents an individual donor. Data are presented as mean for each population (n = 4). Statistical analyses were performed using one-way ANOVA followed by REML (mixed effect model) test. (* *p* < 0.05, ** *p* < 0.01, *** *p* < 0.001). Non-significant differences (*p* ≥ 0.05) are not indicated. Color code: F25 ● F34 ● F36 ● F38 ● F39 ● F49 ● F54 ● F56 ●.

The effect of IGF-1 LR3 on collagen deposition was further evaluated using a larger panel of donors. In agreement with the screening experiments, IGF-1 LR3 significantly increased collagen deposition, whereas β-estradiol did not (Figure 4A). Although β-estradiol supplementation did not enhance collagen content, histological analysis revealed a modest but significant increase in dermal thickness compared to control tissues (Figure 4B). This effect was even more pronounced in tissues cultured in the presence of IGF-1 LR3, which showed the greatest increase in thickness among all conditions tested. No significant difference has been observed in DNA quantification in tissues between conditions (Figure S2). These data support that IGF-1-induced increases in collagen content are not driven by changes in cell number but rather reflect enhanced extracellular matrix production.

### 3.4. IGF-1 Improved the Mechanical Properties of Dermis Reconstructed by the Self-Assembly Method

**Figure 5:**
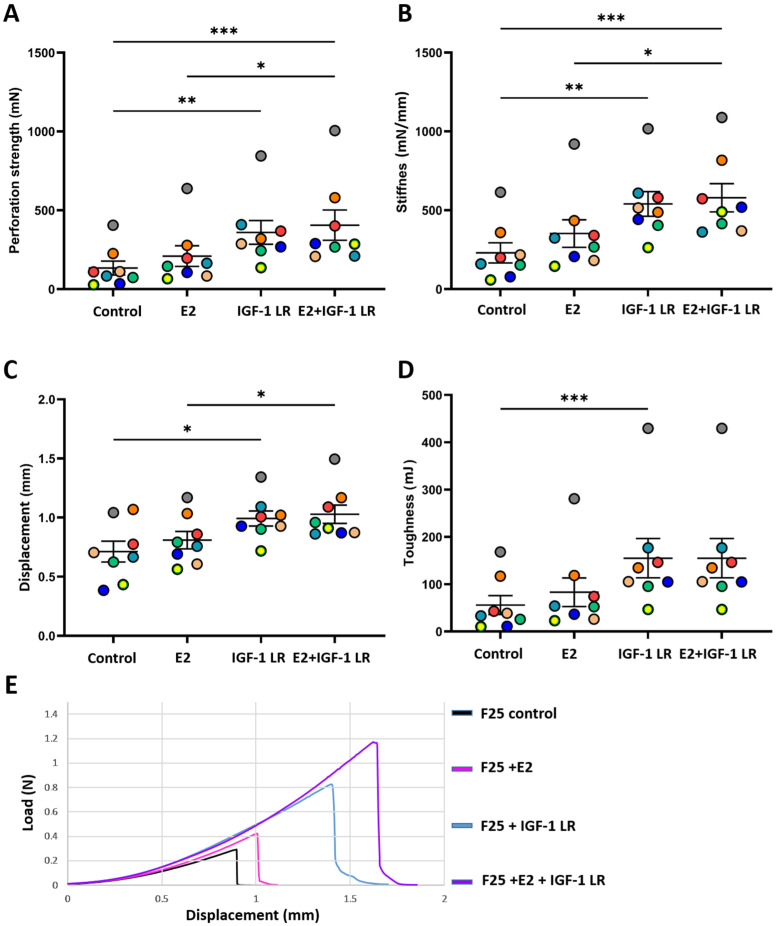
**Improvement of mechanical resistance, stiffness, displacement and toughness by IGF-1 LR3 and estradiol.** Mechanical characterization of reconstructed dermal tissues (28 days-old) treated with estradiol (E2, 10 nM), IGF-1 LR3 (IGF-1 LR, 1 µg/mL), or their combination. (**A**) Perforation strength measured by puncture testing (mN). (**B**) Apparent stiffness derived from stress–strain curves (mN/mm). (**C**) Displacement at break measured during puncture testing (mm). (**D**) Toughness calculated as the area under the stress–strain curve (mJ). (**E**) Representative load–displacement curves illustrating the mechanical behavior of reconstructed dermal tissues under different conditions. Curves are shown for a single donor (F25) and reflect trends consistently observed across donors. Each symbol corresponds to an individual donor. Data are presented as mean ± SD (n = 4). Statistical significance was determined using one-way ANOVA with Friedmann test analysis (*p* < 0.05, * *p* < 0.01, ** *p* < 0.005, *** *p* < 0.001). Non-significant differences (*p* ≥ 0.05) are not indicated. Color code: F25 ● F34 ● F36 ● F38 ● F39 ● F49 ● F54 ● F56 ●.

Mechanical properties of reconstructed dermal tissues were assessed using puncture testing. Perforation strength was significantly increased in tissues cultured with IGF-1 LR3, whereas β-estradiol alone or in combination with IGF-1 LR3 did not result in a significant improvement compared to control tissues (Figure 5A). Similar trends were observed for tissue stiffness (Figure 5B), with a non-significant tendency toward increased stiffness in the β-estradiol condition.

Displacement at break followed the same pattern, with significantly higher values observed in IGF-1 LR3—treated tissues, while β-estradiol did not induce a statistically significant effect (Figure 5C). Finally, tissue toughness was markedly increased in IGF-1 LR3 –supplemented conditions, whereas supplementation with β-estradiol did not significantly alter this parameter (Figure 5D).

**Figure 6:**
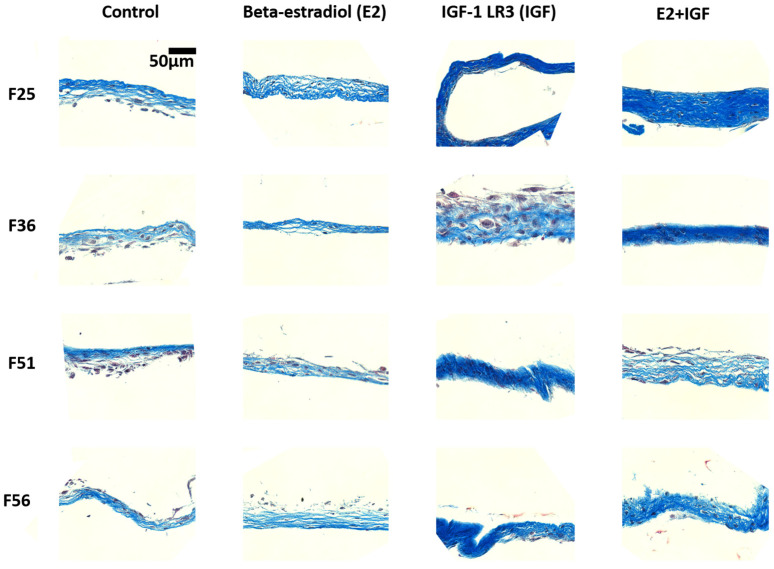
**Histological appearance of reconstructed dermis.** Dermis were reconstructed using the self-assembly method with the standard protocol or with the addition of beta-estradiol (E2), IGF-1 LR3 (IGF-1 LR) or combination of both (E2 + IGF-1 LR). Tissues were stained using the Masson’s Trichrome protocol (collagen fibers are colored in blue). All images are shown at the same magnification and within comparable planes; original images are provided in Supplementary Materials.

Histologies from the tissues tested in Figure 4 and Figure 5 (Figure 6) showed an increase in thickness of tissues produced in culture treated using IGF-1 LR3 and an increase in blue staining density of Masson’s Trichrome staining indicating an increase in collagen.

Together, these results demonstrate that IGF-1 LR3 is a potent enhancer of extracellular matrix deposition and mechanical performance in dermal tissues reconstructed using the self-assembly approach, including when fibroblasts derived from Postmenopausal female donors were used.

## 4. Discussion

The present study sought to identify biochemical strategies capable of enhancing extracellular matrix deposition and mechanical performance of dermal tissues reconstructed using the self-assembly approach with the perspective to produce engineered MUS which serve as an illustrative example of potential use.

Given the well-documented age-associated decline in fibroblast activity, particular attention was paid to donors representative of the population most affected by stress urinary incontinence [34,35]. In this context, the study specifically addresses the age-associated decline in extracellular matrix production by human fibroblasts and identifies IGF-1 signaling as a robust approach to restore collagen deposition and mechanical integrity.

Consistent with previous observations [36], dermal fibroblasts derived from postmenopausal female donors exhibited a significantly reduced capacity to deposit collagen compared to cells isolated from premenopaused donors (Figure 1). This donor-dependent variability highlights one of the main challenges associated with the development of biologically engineered MUS, namely the need to compensate for age- and hormone-related alterations in stromal cell function.

Hormonal supplementation with β-estradiol and progesterone was first evaluated, given the well-established role of estrogens in connective tissue homeostasis and wound healing. In the present model, neither hormone significantly increased collagen deposition (Figure 2A,B), despite a modest increase in tissue thickness observed in β-estradiol–treated conditions (Figure 4B). This dissociation between collagen content and tissue thickness suggests that estrogens may influence the production or organization of other extracellular matrix components, such as proteoglycans or glycoproteins, rather than directly stimulating collagen synthesis. These findings are in line with reports indicating that estrogen effects on stromal tissues are highly context-dependent and influenced by donor age, hormonal status, and local metabolic conditions [37,38,39].

The screening of alternative signaling pathways further emphasized the sensitivity of collagen deposition to cellular metabolic state. Induction of hypoxia using cobalt chloride resulted in a marked reduction in collagen production, which is consistent with the known inhibitory effects of hypoxia on fibroblast collagen synthesis and matrix maturation (Figure S1). In contrast, activation of purinergic signaling through adenosine supplementation did not enhance collagen deposition (Figure 2C) and, when combined with IGF-1 LR3, even resulted in a dose-dependent decrease in collagen content (Figure 3B). These observations suggest that, in the context of the reconstruction of dermis using the self-assembly technique, adenosine-mediated signaling may interfere with anabolic pathways or shift fibroblast activity toward alternative metabolic programs that are not conducive to robust matrix production.

Among all tested conditions, stimulation of the insulin signaling pathway using IGF-1 emerged as the most effective strategy to enhance collagen deposition. IGF-1 LR3 induced a strong and reproducible increase in collagen content in both screening experiments and validation studies performed on a larger donor cohort (Figure 3A and Figure 4A). Importantly, this effect was maintained in fibroblasts derived from women over 50 years of age, indicating that IGF-1 LR3 can partially overcome age-associated declines in extracellular matrix production. The absence of a synergistic effect between IGF-1 and sex hormones (Figure 3B and Figure 4A) further suggests that IGF-1 acts through dominant anabolic pathways that are not substantially amplified by concurrent estrogen or progesterone signaling under the present culture conditions.

When focusing specifically on fibroblasts from older donors, IGF-1 consistently increased collagen deposition in both experimental settings, although the magnitude of the effect differed between Figure 3C and Figure 4A. In Figure 3A, IGF-1 induced a marked increase in collagen deposition under controlled screening conditions, whereas in Figure 4A, the effect appeared more moderate when assessed across a broader panel of donors. This difference in amplitude likely reflects increased inter-donor variability and normalization approaches rather than a true discrepancy in biological response. Importantly, when data are considered relative to their respective controls, IGF-1 still promotes collagen deposition in fibroblasts from older donors, confirming the robustness of the observed effect.

Beyond biochemical outcomes, the increase in collagen deposition induced by IGF-1 LR3 translated into substantial improvements in the mechanical properties of reconstructed tissues. IGF-1–treated tissues exhibited significantly higher perforation strength, stiffness, displacement at break, and toughness, collectively reflecting a more resilient and mechanically robust construct (Figure 5). Notably, β-estradiol supplementation did not significantly enhance these mechanical parameters, either alone or in combination with IGF-1 LR3, despite inducing a modest increase in tissue thickness. These findings underscore the central role of collagen abundance and organization, rather than tissue thickness alone, in determining the mechanical performance of self-assembled tissues.

Although IGF-1 is known to regulate extracellular matrix production through multiple signaling pathways, the precise molecular mechanisms underlying the observed effects in this model were not specifically investigated. Future studies will be needed to better characterize these pathways and to determine whether their targeted modulation could further optimize matrix synthesis in engineered tissues.

Taken together, these results demonstrate that IGF-1 is a potent modulator of stromal extracellular matrix production and a key determinant of mechanical reinforcement in dermal tissues engineered using the self-assembly approach. By restoring collagen deposition and improving biomechanical properties in tissues reconstructed from postmenopausal donors, IGF-1 LR3 supplementation represents a promising strategy to address one of the major limitations currently hindering the clinical translation of biological MUS. More broadly, these findings reinforce the importance of targeting stromal cell metabolism and anabolic signaling pathways to optimize tissue engineering outcomes, particularly in patient populations characterized by age-related or hormone-dependent impairments in matrix synthesis. Nevertheless, in vivo validation will be required to confirm these findings and to assess the behavior of IGF-1 LR3-supplemented MUS in a preclinical context, particularly to rule out any adverse effects such as tissue degradation.

It is important to note that the present study was not designed to generate tissue constructs meeting clinical mechanical requirements. The mechanical characterization was performed on single-layer self-assembled stromal sheets, which are known to exhibit limited mechanical strength. In our standard tissue engineering approach, clinically relevant constructs are obtained by stacking multiple layers, typically three or more, resulting in substantially improved strength. Previous work from our group has shown that such multilayered constructs can lead to constructs reaching force levels that are consistent with values reported in the literature (e.g., ref. [40]).

Accordingly, the objective of the present study was not to produce implantable tissues, but rather to assess whether biochemical modulation with IGF-1 could enhance extracellular matrix deposition and improve mechanical parameters at the level of a single stromal layer. This reductionist approach allows a more precise evaluation of the effect of biochemical factors while minimizing structural confounders associated with multilayer constructs. These observations support the relevance of IGF-1–mediated matrix enhancement for future translational applications, although further validation will be required in fully reconstructed tissues. Importantly, improvements observed at the single-layer level are expected to translate into cumulative gains in multilayer constructs.

The question of whether patient-specific cells are required for tissue engineering applications is indeed context-dependent. In many cases, engineered tissues are produced using allogeneic cells and subsequently decellularized to generate off-the-shelf products. Such approaches represent a well-established and clinically relevant strategy. In the present study, the use of primary human fibroblasts from different donors was primarily intended to investigate inter-donor variability and, more specifically, the impact of aging on extracellular matrix deposition. This approach allowed us to identify IGF-1 as a robust strategy to enhance collagen production across donor profiles, including cells derived from older individuals.

From a translational perspective, several options may be considered. On the one hand, the use of allogeneic fibroblasts with high matrix-producing capacity could facilitate large-scale production under GMP conditions. On the other hand, the development of fully autologous constructs may offer advantages in terms of immunological compatibility and patient acceptance, particularly in the context of biomaterial-free approaches.

While it remains unclear whether living engineered tissues provide superior long-term performance compared to decellularized constructs, the present results support the idea that enhancing matrix production at the cellular level may benefit both strategies. Further studies will be required to determine the most appropriate approach depending on the clinical context.

Beyond its initial application to the development of biologically engineered MUS, the present findings may also have broader implications for other tissues reconstructed using the self-assembly approach [41,42,43]. In particular, the robust effect observed with IGF-1 LR3 supplementation alone, regardless of fibroblast donor age, suggests that modulation of stromal extracellular matrix production may represent a generally applicable strategy to enhance the mechanical performance of self-assembled tissues. While IGF-1 is a potent anabolic factor, its use in the present study was limited to the reconstruction phase and did not result in increased DNA content, suggesting no overt hyperproliferative effect under these conditions.

While the current study focused on dermal fibroblasts in the context of stress urinary incontinence, similar approaches could be relevant for other stromal-based tissue constructs in which extracellular matrix quantity and organization are critical determinants of functionality.

In addition, these observations may also be of interest for tissue engineering strategies relying on temporary biomaterial scaffolds, such as collagen- or fibrin-based hydrogels, where de novo matrix deposition by resident cells is required to progressively replace the scaffold during degradation [44,45,46,47]. In this context, biomaterials designed to locally and gradually release IGF-1 as they degrade could represent a particularly attractive strategy, by sustaining fibroblast anabolic activity over time and promoting organized collagen deposition. Such controlled delivery systems may allow lower overall growth factor doses while maintaining biological efficacy, thereby improving safety and translational potential.

Beyond tissue engineering strategies based on the self-assembly approach, the present findings may also be relevant in an in vivo context. IGF-1 has long been recognized for its role in tissue repair and regenerative processes, notably by stimulating fibroblast activity, collagen synthesis, and matrix remodeling. In this perspective, local and sustained delivery of IGF-1 could be envisioned as a biological adjunct to surgical procedures, with the aim of supporting endogenous tissue reinforcement rather than replacing native structures. Such an approach may be particularly relevant in the context of pelvic floor reconstruction, where promoting host tissue remodeling remains a key determinant of long-term clinical success.

While stress urinary incontinence and mid-urethral sling–related applications provided an important clinical motivation for this work, the primary contribution of the present study lies in the identification of IGF-1 signaling as a powerful modulator of age-associated extracellular matrix decline in human fibroblasts. The use of dermal fibroblasts from postmenopausal donors was intentionally chosen as a biologically relevant model of stromal aging, independently of a specific tissue or device application.

Sex steroid supplementation was evaluated not as a MUS-specific intervention, but rather as a broadly relevant biological strategy, given the known impact of menopause on stromal cell function and connective tissue homeostasis. The absence of a significant effect of estradiol and progesterone on collagen deposition in this model underscores the complexity of hormonal regulation in aged fibroblasts and highlights IGF-1 signaling as a more robust and consistent anabolic pathway.

In the specific context of mid-urethral sling development, these findings support the feasibility of using biochemical modulation to partially overcome age-related deficits in matrix production. However, the relevance of this strategy extends beyond MUS applications and may inform the optimization of a wide range of stromal tissue-engineered constructs intended for aged or hormonally altered patient populations.

Whether such effects can be reproduced across different tissue types, cell sources, or scaffold-based systems remains to be determined and will require dedicated experimental validation.

In addition, increasing regulatory constraints related to safety, together with the well-documented batch-to-batch variability of fetal bovine serum, have driven substantial efforts toward the development of xeno-free culture media [48,49]. In this context, it would be particularly relevant to investigate whether such fully defined media could be supplemented with IGF-1 to support the production of engineered tissues exhibiting mechanical properties compatible with clinical translation or with robust models for fundamental research. Achieving an optimal balance between safety, reproducibility, and functional performance remains a key challenge, but also represents an important opportunity for the broader implementation of tissue-engineered models in both preclinical and clinical settings.

## Data Availability

The raw data supporting the conclusions of this article will be made available by the authors on request.

## Supplementary Materials

The following supporting information can be downloaded at: https://www.mdpi.com/article/10.3390/cells15111023/s1, Figure S1: Effect of cobalt chloride (chemically induced hypoxia) on collagen deposition by dermal fibroblasts; Figure S2: DNA quantitation in dermal fibroblast culture treated with IGF-1 LR and/or estrogen.

## Abbreviations

The following abbreviations are used in this manuscript:

SUI	Stress urinary incontinence
MUS	Mid urethral sling
FDA	Food and Drug Administration
ECM	Extracellular matrix
IGF-1	Insulin like growth factor 1
PBS	Phosphate-buffered saline
DMEM	Dulbecco’s Modified Eagle’s Medium
FBS	Fetal bovine serum
DMSO	Dimethyl sulfoxide
ANOVA	One-way analysis of variance
