# Supplementary material for: IGF-1 Increases Collagen Deposition by Dermal Fibroblasts: Applications for Tissue Engineering
Source: Cells. 2026 Jun 2;15(11):1023. doi: 10.3390/cells15111023 (PMC13257193; doi:10.3390/cells15111023)
Supplement: Supplementary file 1 [file cells-15-01023-s001.zip › cells-4318232-supplementary.pdf]

Supplementary materials

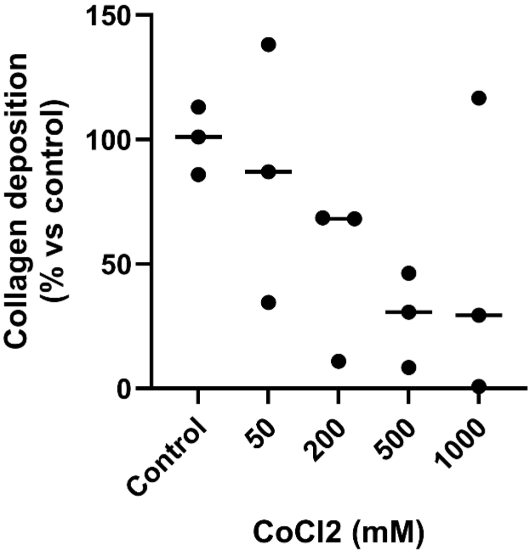

Figure S1. Effect of cobalt chloride (chemically-induced hypoxia) on collagen deposition by dermal fibroblasts. Effect of cobalt chloride ($CoCl_2$) on collagen deposition by dermal fibroblasts cultured using the self-assembly approach. Total collagen content was quantified using Sirius Red staining and normalized to control conditions. N=1 (F34) n=3. Values are expressed as fold change relative to untreated controls. Data represent mean values from 3 independent experiments.

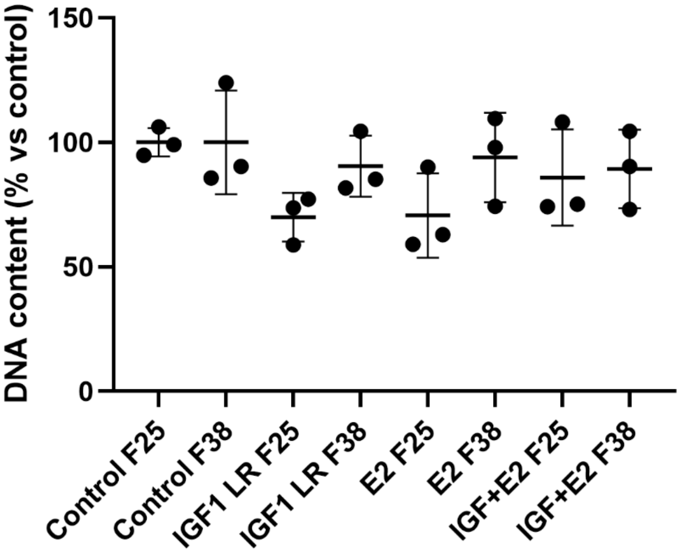

Figure S2. DNA quantitation in dermal fibroblast culture treated with IGF-1 LR and/or estrogen. DNA present in cell culture was quantified using picogreen test and normalized to control conditions N=2 (F25 and F38) n=4. Values are expressed as fold change relative to untreated controls. Data represent mean values from 4 independent experiments. No significant differences were observed between conditions.

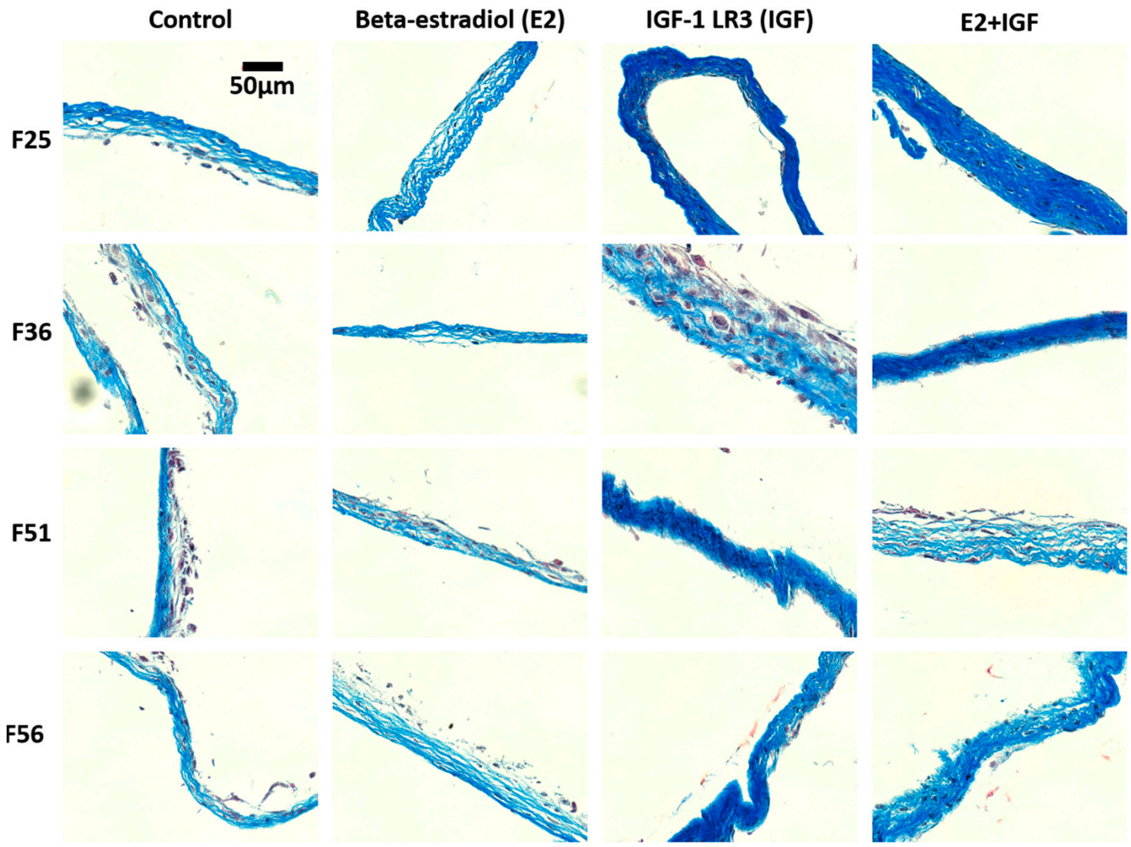

Figure S3. Original images for Figure 6
